# A Mixed Methods Evaluation of the Statutory Duty of Candour in Victorian Health Services: Study Protocol

**DOI:** 10.1111/hex.70180

**Published:** 2025-02-12

**Authors:** Reema Harrison, Corey Adams, Nabila Binte Haque, Jennifer Morris, Liat Watson, Kristiina Siiankoski, Ashfaq Chauhan, Thrivedi Sesha Sai Danthakani, Sarah Ameen, Peter Hibbert, Elizabeth Manias, Nicole Youngs, Lanii Birks, Ramesh Walpola, Sarah Fischer, Jeffrey Braithwaite

**Affiliations:** ^1^ Australian Institute of Health Innovation Macquarie University North Ryde New South Wales Australia; ^2^ Safer Care Victoria Melbourne Victoria Australia; ^3^ School of Nursing and Midwifery Monash University Frankson Victoria Australia

**Keywords:** adverse event, apology, incident disclosure, patient safety, statutory duty of candour

## Abstract

**Background:**

A statutory duty of candour (SDC) was introduced in 2022 in the Australian state of Victoria to increase openness and honesty with patients and families about healthcare adverse events. SDC requires each healthcare service entity by law to provide the patient or their family or carer who experiences an adverse event with; a written account of the facts, an apology, a description of the health service's response to the event, and the steps being taken to prevent reoccurrence. This research aims to evaluate the impacts of SDC in the 2 years since its implementation.

**Design:**

A mixed‐methods sequential design will be employed, comprising a document literature review, document analysis, survey and interview data from patients, families and health service staff between 2024 and 2026.

**Discussion:**

By conducting an evaluation of the impacts of SDC within 2 years of its implementation in Victorian health settings, this research will provide the first independent evidence of how the SDC is being used and affecting healthcare experiences. This research will use evaluation criteria from the UK's Duty of Candour review to gather data that can be compared with UK findings and the disclosure experiences of patients in New South Wales, Australia. Our findings will provide a vital contribution to the sparse evidence base about the SDC and its application in healthcare settings internationally.

**Patient or Public Contribution:**

Three members of the public (JM, LW and KS) were involved in the design of the research proposal, reviewing and contributing to the ethics protocol, the protocol paper as authors and the protocol for the systematic review that has been developed as a basis for this research. These collaborators will contribute to be involved in all aspects of the research as part of the project team.

## 1 Background

Patients and families affected by physical or psychological harm arising from their healthcare benefit from open and honest communication by healthcare staff about the events that have occurred [1, 2, 3]. Several health systems have adopted frameworks to encourage best practice, inclusive of transparent communication and apology every time an adverse event occurs. This is referred to as *‘incident disclosure’* or *‘open disclosure’* [4, 5]. In Australia, the Open Disclosure Standard was released in 2003. Following a review of the Standard in 2011, it was replaced by the Open Disclosure Framework endorsed nationally in 2012 [4]. Similarly, in the UK, the Being Open Framework was first released in 2002, and updated in 2012 [1].

Despite advancements in knowledge about the gains of open and honest communication and apology, and the adoption of incident disclosure frameworks and management policies, limited progress was demonstrated in the rate or quality of disclosure [3]. Fear of punitive action associated with legal implications of providing an apology and an absence of mandatory requirement for disclosure were cited as key barriers [1, 6]. With lack of disclosure or poor quality disclosures continuing to cause distress among patients and families affected by healthcare adverse events, consumer groups advocated for a statutory duty of candour (SDC) alongside disclosure frameworks to enhance their implementation and foster an open and honest culture [7]. The Duty requires each health service entity by law to provide the patient or their family or carer who experiences an adverse event with; a written account of the facts, an apology, a description of the health service's response to the event, and the steps taken to prevent reoccurrence. The threshold for an adverse event (events in which a patient is harmed by the healthcare they receive) to meet the SDC requirement varies between countries, but the focus is primarily on events that have caused patient harm [8, 9].

SDC or similar legislation and policies have been introduced in several countries over the past 10 years: England, Wales, Scotland, Ireland, Australia, Germany, Canada; yet its impacts in improving the management of adverse events for patients and families or the accountability of healthcare providers have not been evaluated until now [10]. The UK Department of Health and Social Care recently launched a review of the Duty of Candour to establish its compliance and enforcement in all health and social care providers that the Care Quality Commission (CQC) regulates. The review comes 10 years after the introduction of SDC in UK health settings [9]. Evidence gathering ceased in late May 2024 and a report of findings is forthcoming.

In Australia, SDC was introduced in one of the eight Australian states and territories rather than as a national requirement. As part of a wider culture of change in health services across the Australian state of Victoria, SDC and protections for adverse event reviews were recommended. The SDC was introduced by the Victorian Health Legislation Amendment (Quality and Safety) Act 2022 (the Act) to foster an open and honest culture by elevating existing obligations of open disclosure and apologies. In Victoria, SDC applies when moderate or severe harm occurs to patients. The Act introduced protections for adverse event reviews called serious adverse patient safety event (SAPSE) reviews, to encourage open and frank discussion between health services and consumers in such circumstances. Under the new legislation, if a patient experiences a SAPSE, the health service entity has a legal requirement to provide the patient or their family or carer with; a written account of the facts, an apology, a description of the health service's response to the event, and the steps being taken to prevent reoccurrence.

Independent evaluation of the implementation and impacts of SDC in all Victorian health settings is being conducted by academic researchers at the Australian Institute of Health Innovation supported by consumer advisors to the project. A three‐phase research programme has been established that will evaluate SDC's impact within the 2 years since its implementation in Victorian health settings, with ethical approval granted, and data collection is set to begin in late 2024.

### 1.1 Aim

The overall aim of this research is to establish knowledge, experiences and impacts of SDC on the management of SAPSE in Victorian health services from the perspectives of consumers, the public, and healthcare staff. The aim will be addressed by a series of studies that achieve the following objectives:

1. Establish how SDC has been implemented in the management of adverse patient safety events and compliance with the legislation.
2. Determine knowledge, awareness and understanding of SDC among healthcare staff, consumers and the public.
3. Determine the perceived effectiveness of SDC and its effects on patients, carers, staff and service delivery resulting from its enactment.
4. To assess the impact of healthcare service's accountability in learning from adverse events and the effectiveness of the SDC in promoting transparency and communication between healthcare staff and patients within 2 years of its implementation.
5. Identify the implementation challenges and barriers experienced by healthcare staff.

## 2 Methods

### 2.1 Research Design

**Figure 1:**
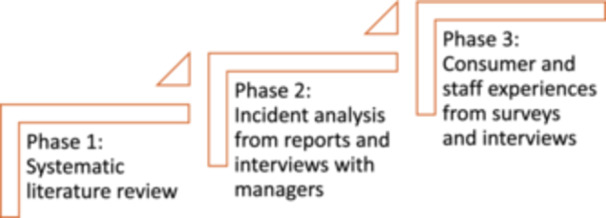
Research programme overview.

A mixed‐methods sequential design will be employed comprising of literature review, document analysis, survey and interview data from patients, families and health service staff. The overall completion of the project (Figure 1) is expected to take 2 years (2024–2026).

### 2.2 Phase 1: Systematic Literature Review

#### 2.2.1 Design

A systematic review and narrative synthesis will be conducted according to the recommendations from Cochrane and reported according to the Preferred Reporting Items for Systematic Reviews and Meta‐Analyses (PRISMA) statement [11]. The review has been registered in the PROSPERO database CRD42024575710.

#### 2.2.2 Review Aim

To establish how SDC has been implemented in the management of patient safety events and the effects on patients, carers, staff and service delivery resulting from its enactment.

#### 2.2.3 Eligibility Criteria

For articles to be eligible for inclusion they require participants who were stakeholders in SDC including patients and/or families, healthcare or medico‐legal staff. Articles will be required to report primary or secondary data on the use of SDC (defined as a legal requirement for healthcare staff and/or their organisations to respond in a transparent way) in response to a patient safety incident (defined as instances in which there was potential or actual healthcare‐associated patient harm given the different thresholds for legal duties to be enacted internationally). Patient safety event represents a broader inclusion criterion than just adverse event to reflect the varying thresholds for SDC internationally. Case studies of incidents in contexts in which SDC was enacted will be eligible. Articles from countries in which SDC has been implemented will be eligible. No restrictions will be applied on study design. No comparator will be required. Eligible outcomes will be the experience of SDC implementation among healthcare staff or consumers of SDC, patient and carer, staff or service delivery outcomes. Additionally, articles will be excluded if they focus on compensation and resolution, no‐fault systems, apology laws, or other programmes that are not explicitly related to SDC, or if they describe SDC without providing outcome data.

#### 2.2.4 Outcomes

Primary outcomes of interest are implementation outcomes relating to use of SDC, and data on patient and carer, staff or service delivery outcomes resulting from its application. Implementation outcomes eligible for inclusion will be defined according to Proctor et al. [12] as outcomes reporting acceptability, adoption, appropriateness, cost, feasibility, fidelity, penetration or sustainability of SDC in healthcare provision. Patient or carer outcomes will be defined as personal, professional, or health (physical or psychological) outcomes relating to the patient or carer following experience with SDC. Staff outcomes will be defined as personal, professional, or health (physical or psychological) outcomes relating to staff following experience with SDC. Service delivery outcomes will be defined as workforce, procedural, policy, financial, service utilisation or practice change outcomes relating to the use of SDC.

#### 2.2.5 Search Methods for Identification of Studies

A search strategy has been developed (Supporting Information S1: File S1) comprised of relevant keyword and MeSH terms for adverse patient safety events, disclosure SDC from countries that had enacted legal requirements. The search strategy will be applied to electronic databases (MEDLINE, Embase, CINAHL, Web of Science, Sage Journals and ProQuest) and grey literature. The websites of public health system and service organisations from countries in which SDC has been implemented and that have a role in SDC will be identified through online searches. Keywords identified in our search strategy (Supporting Information S1: File S1) will be applied to the websites of eligible organisations. We will contact the authors of all unobtainable studies or studies with missing data. No restrictions on language or publication date will be applied. Two reviewers will independently use citation pearl growing to hand search references of eligible studies and reviews identified from the search. Authors of study protocols will be contacted if the study status indicated findings may be available, but no published work is identified.

#### 2.2.6 Study Selection, Data Extraction and Risk of Bias

Covidence software will be used to enable independent screening and the identification of conflicts to resolve. Two reviewers will independently screen titles and abstracts of identified studies and articles. These reviewers will then independently assess the full text of articles and seek to achieve consensus on eligibility through discussion. Two further reviewers will independently extract data and assess risk of bias using MetaQAT (20). Disagreements between reviewers will be resolved by consulting a third reviewer.

#### 2.2.7 Data Synthesis

The Synthesis Without Meta‐Analysis (SWiM) guideline will be used to guide the reporting of this systematic review, assuming that a meta‐analysis is not likely to be feasible if there is heterogeneity in the research designs, article types and outcomes (21). Findings will be grouped for the narrative synthesis by the primary outcomes of the review (implementation, patient and family, staff and service delivery outcomes) due to the intended outcomes of SDC. To conduct the narrative synthesis (22), tabulation of study features will be undertaken by two reviewers and used to develop a preliminary synthesis of the included studies. Relationships in the data will be explored in relation to each of the outcome groupings by two reviewers who will employ data charting and categorisation to develop a richer view of the findings. Thematic summaries will then be developed and discussed with the wider review team to develop the final synthesis of the evidence relating to each outcome. Critical reflection on the risk of bias assessments and the Grading of Recommendations Assessment and Evaluation (GRADE) approach (23) will be employed to make judgements about the certainty of evidence if feasible.

### 2.3 Phase 2: Incident Analysis

#### 2.3.1 Design

Mixed methods synthesising analysis of incident data with interviews will be used to assess the impacts of SDC on health service's accountability and specifically their accountability in learning from adverse events in Victoria within 2 years of implementation of the SDC.

#### 2.3.2 Aim

To assess the impact of health service's accountability in learning from adverse events and the effectiveness of the SDC in promoting transparency and communication between healthcare providers and patients within 2 years of its implementation.

#### 2.3.3 Incident Data Analysis

##### 2.3.3.1 Data Source

Incident management data will be sought from the Victorian Health Incident Management System (VHIMS) and provided by Safer Care Victoria on behalf of the Victorian Department of Health, from Victorian public health services for the 12‐month period from November 2021 to November 2022 before SDC and for the 12‐month period from November 2022 to November 2023 following the SDC implementation. e‐Health Victoria estimates 120,000+ incidents from 1 November 2021 to date with the incident fields shown in Supporting Information S1: File S2.

##### 2.3.3.2 Data Analysis

The status of the VHIMS Minimum Data Set (MDS) open disclosure field will be assessed before and after SDC implementation. We will stratify the analysis by its incident severity rating (ISR), healthcare service and peer group, and incident type, and other VHIMS fields. The analysis will identify characteristics (e.g., combinations of ISR, health service, and incident type) of incidents where there have been significant changes in open disclosure status and explore trends in ISR ratings since the implementation of SDC. The analysis will also identify incident characteristics associated with significant outliers of high or low open disclosure status. This quantitative analysis will then guide a focused qualitative analysis of sets of incidents to understand the reasons for the results.

#### 2.3.4 Semi‐Structured Interviews

##### 2.3.4.1 Sample

Healthcare managers aged 18 at a range of levels will be recruited. Eligible healthcare managers will have experience of working in Victorian health services with responsibility for quality and safety of health care, including before SDC implementation. Eligible individuals will be invited to participate in individual semi‐structured interviews to explore their perceptions of the impacts of SDC on accountability. Recruitment will target individuals from Victorian health services and state agencies primarily, in addition to those from federal agencies and further states to provide a comparative analysis. We will recruit approximately 20 individuals, stratified by state and federal levels.

##### 2.3.4.2 Procedure

Results of the incident analysis and the literature review will be presented to interviewees in a summary format. The interviewees will be guided through a semi‐structure schedule to facilitate them to provide further interpretation of this evidence to provide a rich understanding of the health service's accountability and learning from adverse events in Victoria within 2 years of implementation of the SDC.

##### 2.3.4.3 Data Analysis

Interview data will be subject to a thematic qualitative analysis conducted by two researchers, which will be undertaken using an inductive and deductive approach via the Framework method [13]. An organisational accountability framework will be used to examine the data [14].

### 2.4 Phase 3: Consumer and Staff Experiences

#### 2.4.1 Design

Mixed methods evaluation synthesising survey and interview data from patients, families and health service staff.

#### 2.4.2 Aims

(1) To determine knowledge, awareness and understanding of SDC among staff and consumers; (2) to establish the perceived effectiveness and impacts of SDC on consumers and staff; and (3) to identify the implementation challenges and barriers experienced by healthcare providers.

#### 2.4.3 Sample

We aim to recruit 300 consumers and 300 staff to complete surveys, with approximately 25 interviews to be conducted in follow‐up with each of the two cohorts (staff and consumers): totalling approximately 50 interviews. A multi‐channel approach to recruitment will enable the identification of consumers and staff who have accessed a range of service types and locations in Victoria who have diverse characteristics. We will channel recruitment via Safer Care Victoria healthcare staff and consumer networks, consumer groups and support organisations, in addition to using social media, health professional networks and colleges as well as the research team's extensive network.

*Data collection tools:*

i. Patient survey: An existing validated 15‐item scale used within a survey of > 7500 patients and families in NSW (6) to report their awareness, understanding, experiences and perceptions of incident disclosure in 2019, was used as the basis for the design of the present research so that evidence of the results reported by Victorian patients and families can be compared with those of patients and families in NSW. Items have been adapted where required to evaluate awareness and understanding of the SDC, and the extent to which it has improved the management of SAPSE. Additional items were added to align with the questions posed of consumers in the UK SDC review to enable comparisons to be drawn.
ii. Staff survey: The existing validated 15‐item scale used for the patient survey was also used as the basis for the staff survey content. Items were reframed to reflect a provider perspective in relation to experiences of incident management in the past 2 years. Specifically, items were revised or added to capture the perceived impacts of SDC on practice. Additional items were added to align with the questions posed of staff in the UK SDC review to enable comparisons to be drawn.
iii. Consumer and staff interviews: Follow‐up interviews with patients, families and staff were guided by an interview schedule used in the UK Being Open policy evaluation (7). A core topic guide was developed and will be applied with patients, families and health service staff that explored these stakeholder's awareness and understanding of incident management, their personal experiences of its implementation and its perceived effectiveness and impacts. This existing topic guide has been reviewed and revised to refine the scope relative to the implementation of SDC specifically.

#### 2.4.4 Procedures

To promote anonymity and confidentiality, the survey will be conducted online using MQ Qualtrics software. An embedded link to the survey will be included in the project recruitment materials so that this can be directly opened to enable survey responses via computer or mobile device. Contact information for the project team will be provided so that paper‐based copies or a translated version can be requested. At the end of the survey, participants will have the option to either provide their contact details to participate in an interview or to contact the project team directly to request an interview. Interviews will be conducted by a member of the project team, all of whom are experienced in conducting interviews about complex and sensitive health service topics. Interviews will be conducted using Microsoft Teams to enable recording, transcription and these data to be securely stored for analysis in the Macquarie University OneDrive.

#### 2.4.5 Data Analysis

Frequencies and percentages will be calculated from the survey data using Stata 18 (Stata Corp, College Station, TX, USA). Pearson Chi‐squared tests will be used to assess the significance of differences between groups, for example those who have and have not experienced an incident, patients and carers, staff from different professions. A significance level of 0.05 will be used for analyses. Interview data will be analysed inductively and deductively using the Framework approach [13]. Qualitative data will be managed using NVivo software version 12 (QSR International, Melbourne, Vic, Australia). Two researchers will independently read the transcripts and conduct a thematic analysis to identify key concepts, ultimately developing these into themes through discussion with the wider team, including consumers. Groups of themes will then be merged into categories and labelled. Equity of impacts will be explored in the analysis in terms of patient, provider and service characteristics. A third researcher will review the final categories and themes that are developed before final analysis.

#### 2.4.6 Evidence Synthesis

Framework synthesis will be used to integrate Phase 1–3 findings to address the research aim [15]. Data will be indexed to synthesise key information relevant to accountability among health services, considering implementation of SDC, settings, incident types and populations.

## 3 Expected Outcomes

By conducting an evaluation of the impacts of SDC within 2 years of its implementation in Victorian health settings, this research will provide the first independent evidence of how the SDC is being used and affecting healthcare experiences. Specifically, the following new insights will be reported:

- the *awareness* and *understanding* of the SDC among patients and their families within 2 years of its implementation;
- the *impact* of the SDC on health services: accountability, the impact of health service's accountability in learning from adverse events, on patient experience of the management of SAPSE;
- the *effectiveness* of the SDC in promoting transparency and communication, improving patient safety culture and fostering a just culture between healthcare providers and patients;
- the *challenges and barriers* faced by healthcare providers in implementing the SDC within the first 2 years of its implementation.

In drawing upon existing tools for measurement, this research will also provide data that can be compared with findings from the UK Duty of candour review and the experiences of disclosure among patients in NSW. Our findings will provide a vital contribution to the sparse evidence base about the SDC and its application in healthcare settings internationally.

## 4 Patient and Public Involvement

The design and conduct of this research places patients and the public at the centre as essential collaborators. Three members of the public (JM, LW and KS) were involved in the design of the research proposal, reviewing and contributing to the ethics protocol, the protocol paper as authors and the protocol for the systematic review that has been developed as a basis for this research. These collaborators will continue to be involved in all aspects of the research as part of the project team. The active involvement of patients and the public as essential collaborators in this research ensures that the findings will have broader relevance, not only to academic research but also to patient‐centred care. With public contributors playing a key role in the design of the research proposal, ethics protocol, and systematic review, the study is well‐positioned to influence clinical practice and inform future research within the field. Their continued participation throughout the project will further enhance its real‐world impact.

## 5 Ethics, Data Storage and Protection

The Human Research Ethics Committee at Macquarie University has approved this study (Project ID 520241807758893; Reference No: 18077). We will obtain implied consent from participants who complete the survey and written informed consent from those who complete interview research. We will ensure participants’ confidentiality and anonymity throughout the study, including during data gathering, management, analysis and reporting processes. All data from this study will be securely managed and protected, and only the researchers involved in the study can access them. We will store electronic data, such as audio recordings and survey responses, on the Macquarie University OneDrive, which is a secure, password‐protected platform that requires two‐factor authentication. Participant consent forms will be stored separately to their data. It is not anticipated that the data will be used for any other purpose, and a new ethics application will be made should there be a proposal to use the data for another purpose. The data will be destroyed per ethical requirements 7 years after the project's completion.

## 6 Projected Outputs and Dissemination Plans

In the absence of evaluative research about the impacts of SDC internationally, and in light of the unfolding UK review of SDC, dissemination of project outputs using broad and accessible approaches is vital. We anticipate producing outputs in formats suitable for academic, consumer, clinical and policy audiences relating to each of the three phases of the research. Beyond open access publication of findings in scientific journals and their presentation at scientific meetings, findings will be communicated via plain language summaries on the Macquarie University project page, infographics posted on social media to reach international stakeholders, and via a report to Safer Care Victoria that will be the basis for their communication of findings to policy and practice decision‐makers. In presenting the work, we will target conferences and meetings that capture a cross‐section of consumer, clinical and policy delegates internationally who share interests in healthcare quality enhancement.

## 7 Anticipated Strengths and Challenges

Being the first academic research project to evaluate the use and impacts of SDC internationally is a core strength of the work. The forthcoming evidence will provide new insight into how well SDC is understood by both patients and health professionals, but also how it is being applied in practice in Victorian health services. By analysing instances of SAPSE, coupled with literature review, survey and interview data, we will be able to provide a rich and expansive analysis of SDC in practice. With reference to other research conducted to date about open disclosure and experiences of healthcare adverse events, challenges in obtaining the sample required and ensuring that respondents have a shared understanding of what constitutes an adverse event, a requirement for disclosure and specifically for SDC will be ongoing. Working in partnership with Safer Care Victoria provides support for promoting and attracting participants to engage with the research. We will draw upon approaches we have previously used with patients and staff to clarify terms and concepts as we gather data. In speaking with people about adverse events occurring in healthcare, we will also need to support participants with difficult conversations and providing psychological support. Our experience as a team in researching this area equips our researchers with the skills and awareness to navigate these experiences appropriately. The research may be constrained by possible confounding changes in the health system during the periods of study on the incident analysis such as COVID‐19, response rate and representativeness of samples in the Phase 3 survey, and by the limitations of the design in establishing effectiveness of SDC on patient, clinician and health system outcomes.

## 8 Discussion

Experiencing an adverse patient safety event can have life‐long consequences for the patients, families and healthcare staff involved [1, 16]. Central to their recovery, and the ability of healthcare providers to learn and make improvements in care, is open and honest communication between these parties about what has occurred and a genuine apology [1, 2]. SDC is considered a critical tool to ensure healthcare providers take the appropriate action in the event of a SAPSE; it has been the subject of extensive campaigns and discussion internationally for more than 10 years [7]. Evaluating the understanding, use, and impact of SDC in this research is a crucial step in determining whether SDC is being implemented as intended and achieving the desired outcomes in managing adverse events. This new knowledge will inform healthcare providers, regulators and government agencies about gaps in knowledge or implementation, and of the target areas for action to ensure that SDC is effectively implemented in the interests of safe and high‐quality patient care.

## Author Contributions

**Reema Harrison:** conceptualisation, investigation, funding acquisition, writing–original draft, writing–review & editing, supervision, methodology. **Corey Adams:** conceptualisation, funding acquisition, investigation, writing–original draft, supervision, methodology, writing–review & editing. **Nabila Binte Haque:** investigation, formal analysis, writing–review & editing. **Jennifer Morris:** conceptualisation, methodology, writing–original draft. **Liat Watson:** conceptualisation, writing–original draft, methodology. **Kristiina Siiankoski:** Conceptualisation, Writing–original draft, Methodology. **Ashfaq Chauhan:** Conceptualisation, investigation, funding acquisition, writing–original draft, methodology, supervision. **Thrivedi Sesha Sai Danthakani:** writing–review & editing, formal analysis, investigation, methodology. **Sarah Ameen:** investigation, methodology, writing–review & editing. **Peter Hibbert:** methodology, investigation, conceptualisation, funding acquisition, writing–original draft, supervision. **Elizabeth Manias:** supervision, conceptualisation, investigation, funding acquisition, writing–original draft, methodology. **Nicole Youngs:** conceptualisation, investigation, funding acquisition, writing–original draft, methodology, supervision. **Lanii Birks:** conceptualisation, investigation, funding acquisition, writing–original draft, methodology, supervision. **Ramesh Walpola:** supervision, conceptualisation, investigation, funding acquisition, writing–original draft, methodology. **Sarah Fischer:** conceptualisation, investigation, writing–original draft, methodology. **Jeffrey Braithwaite:** conceptualisation, investigation, writing–original draft, methodology, supervision, funding acquisition.

## Conflicts of Interest

Liat Watson, Kristiina Siiankoski, Nicole Youngs, Lanii Birks, and Sarah Fischer are employed by Safer Care Victoria who are the research funders. The other authors declare no conflicts of interest.

## Supporting information

Supporting information.

Supporting information.

## Data Availability

Data being collected through this research will be made available in accordance with the conditions of the ethical approval obtained for the research.
