# Supplementary material for: A Mixed Methods Evaluation of the Statutory Duty of Candour in Victorian Health Services: Study Protocol
Source: Health Expect. 2025 Feb 12;28(1):e70180. doi: 10.1111/hex.70180 (PMC11815560; doi:10.1111/hex.70180)
Supplement: Supplementary file 1 — Supporting information. [file HEX-28-e70180-s002.docx]

Database(s): **Ovid MEDLINE(R) ALL**Search Strategy:

**SDC**

**22 May 2024**

| **#** | **Searches** | **Results** |
| --- | --- | --- |
| 1 | ((duty adj3 candour) or (statutory adj3 candour) or open disclosure).mp. |  |
| 2 | 1 and (legal or legislat* or statutory or law or regulat* or mandat* or compulsory).mp. |  |
| 3 | limit 2 to (english language and yr="2010 -Current") |  |
| 4 | (exp medical errors/ and exp disclosure/) or sentinel event*.mp. or critical incident*.mp. or patient safety event*.mp. or patient safety incident*.mp. |  |
| 5 | 4 and (legal or legislat* or statutory or law or regulat* or mandat* or compulsory).mp. |  |
| 6 | 5 and (exp united kingdom/ or exp australia/ or england.mp. or wales.mp. or scotland.mp. or ireland.mp. or australia.mp. or victoria.mp. or queensland.mp. or nsw.mp. or new south wales.mp.) |  |
| 7 | limit 6 to (english language and yr="2010 -Current") |  |
| 8 | 3 or 7 |  |
