# Supplementary material for: A Mixed Methods Evaluation of the Statutory Duty of Candour in Victorian Health Services: Study Protocol
Source: Health Expect. 2025 Feb 12;28(1):e70180. doi: 10.1111/hex.70180 (PMC11815560; doi:10.1111/hex.70180)
Supplement: Supplementary file 2 — Supporting information. [file HEX-28-e70180-s001.docx]

**Supplementary file2 - Incident fields**

***Data scope (inclusions/exclusions):****Victorian Healthcare Incident Management System data set*

***Date range:****1 November 2021 to current*

***Data aggregations:****Nil*

***Fields provided:***

- *Organisation ID*
- *Health service type (or peer group)*
- *Incident ID*
- *Incident date:*
  - *Year*
  - *Month*
- *Incident type: Clinical incidents only*
- *Incident severity: ISR 1 and ISR 2 only*
- *Event type*
- *Open disclosure: Yes/No/Not applicable*
- *Patient gender*
- *Patient age*
